# Rheological Behavior of an Aqueous Suspension of Oxidized Carbon Nanohorn (CNHox)

**DOI:** 10.3390/nano14151247

**Published:** 2024-07-25

**Authors:** Ayumi Moteki, Motoyoshi Kobayashi

**Affiliations:** 1 Graduate School of Science and Technology, University of Tsukuba, 1-1-1 Tennodai, Tsukuba 305-8572, Ibaraki, Japan; 2 Institute of Life and Environmental Sciences, University of Tsukuba, 1-1-1 Tennodai, Tsukuba 305-8572, Ibaraki, Japan

**Keywords:** carbon nanomaterials, surface roughness, aggregation−dispersion, Kreiger-Dougherty equation, porous particle

## Abstract

Oxidized carbon nanohorn (CNHox) a carbon nanomaterial that has attracted attention due to its unique material properties. It is expected to be applied in various areas like cancer treatment, gene-expression technology, fluids with high thermal conductivity, lubricants, and so on. While the rheological measurements of suspensions provide information on the effective size and interactions of suspended particles, the rheological behaviors of aqueous suspensions of CNHox have never been systematically investigated. To clarify the rheological behaviors of aqueous suspensions of CNHox, their viscosity and dynamic viscoelasticity were measured with changing particle concentration and salt concentration. The viscosity of a CNHox suspension showed yield stress at low shear rates and showed shear-thinning behavior with increasing shear rates. The viscosity of 5 weight % CNHox suspensions was comparable to that of 60 weight % silica suspensions. This high viscosity at a low CNHox concentration is probably due to the porous structure and large effective volume of the CNHox particle. The estimated effective volume of CNHox calculated by the Krieger−Dougherty equation was 18.9 times larger than the actual volume calculated by the mass concentration and density. The dependence of rheological behavior of the CNHox suspension on salt concentration was weak compared to that of the colloidal silica suspension. This weak dependence on salt concentration may be due to the roughness of the particle surface, which would weaken the effect of electric double-layer interactions and/or van der Waals interactions between particles. These rheological behaviors of the aqueous suspension of CNHox shown in this research will be useful in efforts to improve the efficiency of its utilization for the various applications.

## 1. Introduction

Carbon nanomaterials such as carbon nanotubes [1], graphene [2], and fullerenes [3] are expected to be applied in many fields due to their strength, large specific surface area, high conductivity, and thermal conductivity. Carbon nanohorn (CNH) is a carbon nanomaterial [4,5] produced by CO_2_ laser ablation of graphite [4]. CNH can be obtained in large quantities at high purity without using catalysts [6], and its peroral toxicity is quite low [7]. CNH exists in dahlia-like irreversible aggregates about 80–100 nm in diameter that consist of thousands of cone-shaped horns [4,8]. CNH has high specific surface area, high thermal conductivity, and good dispersibility in various organic solvents [8,9].

**Figure 1:**
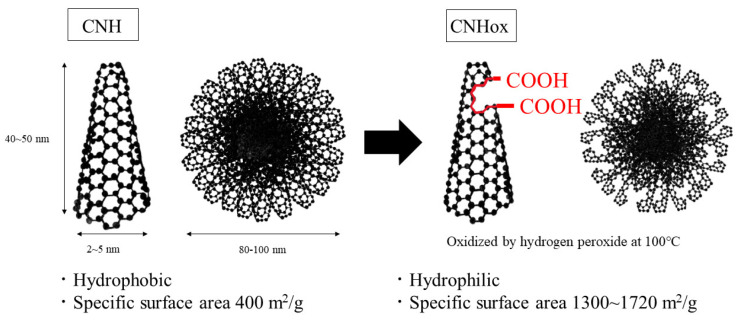
Schematic image of the structure and features of carbon nanohorn (CNH) and oxidized CNH (CNHox). CNHox has chargeable groups on its surface [9,11,12].

The wall of CNH particles can be partly opened by the oxidation of CNH through methods such as heat treatment with O_2_ or CO_2_ [10]. CNH with open holes in the walls is referred to as CNHox [6]. Schematic images of CNH and CNHox are shown in Figure 1. The oxidation opens the closed tips of CNH, which gives CNHox larger specific surface area [10] and better dispersibility into water because chargeable groups such as COOH are present at the edges of the holes [9,11,12]. CNHox has various advantages such as high specific area and better dispersibility, in addition to the merits associated with all carbon nanomaterials. Thus, it is worthwhile to systematically study its properties and potential applications. So far, studies have reported on the application of CNHox in various areas such as cancer treatment, gene expression, fluids with high thermal conductivity, lubricants, and so on [13,14,15,16,17,18,19]. Now, CNHox is recognized as a notable carbon nanomaterial.

Generally, nanoparticles and colloidal particles are dispersed in a solvent and handled as suspensions. However, the use of suspensions containing solid nano/colloidal particles would lead to many problems such as aggregation, sedimentation, and high risk of clogging. Therefore, to work with suspensions, it is essential to identify the rheological behaviors of those suspensions. Suspensions generally show complex rheological behavior due to various factors.

Factors affecting the rheology of a suspension include the structure, surface roughness, and porosity of suspended particles. Generally, the surface roughness of the particles affects the rheology of the suspension [20,21,22,23,24]. Concentrated suspensions of rough particles tend to show more complex rheological behaviors at lower particle concentrations compared to concentrated suspensions of smooth particles, such as high viscosity [20,21], shear-thickening behavior at lower critical shear rates [21,22,23], jamming [23], and small maximum packing fractions [20]. This last is likely due to the interlocking of surface asperities [20,23,24]. The surface of CNHox is considered to be rough because horns stick out of the dahlia-like aggregates [4]. Therefore, a suspension of CNHox with a lower solid concentration is expected to behave like a concentrated suspension. As for the effect of the porousness of the particles on the rheology of the suspension, it is thought that the effective volume of these particles in suspension is large because the solvent in each particle’s pores behaves as a part of the particle. The increase in the effective volume induces an increase in the suspension viscosity [25]. The structure of CNH has been reported to include pores in the horn [26], and CNHox has a large number of holes compared to CNH. Therefore, the dahlia-like and irreversible aggregates of CNHox are expected to behave as porous nanoparticles and the viscosity of a CNHox suspension is expected to be large.

Another factor contributing to the complexity of the suspension’s rheology is the dispersion−aggregation behavior of particles in suspension. The dispersion−aggregation behavior of nano/colloidal particles in suspension is mainly determined by the van der Waals attractive force and electric double layer (EDL) repulsive force. The sum of the van der Waals and EDL interaction potentials between particles forms the basis of the Derjaguin−Landau−Verwey−Overbeek (DLVO) theory [27,28]. The dispersion−aggregation behavior of many colloids and nanoparticles such as colloidal silica [29], cellulose nanofibers [30], and allophane [31] follows the DLVO theory. The aggregation−dispersion of CNHox in aqueous solutions also follows the DLVO theory, where the aggregation is induced by increasing the salt concentration and reducing the magnitude of the zeta potential [32]. Also, the suspension of colloidal silica particles, which follows DLVO theory, is affected by the dispersion−aggregation of particles, which is controlled by the change in salt concentration [33]. In the same way, the rheology of a CNHox suspension is also expected to be affected by the change in salt concentration through the dispersion−aggregation behavior of CNHox.

The above features of CNHox and suspension rheology lead us to expect that the rheological behavior of the CNHox suspension must be complex. However, systematic research on the rheological behavior of a CNHox suspensions of CNH and CNHox is lacking so far. Selvam et al. [15] examined the rheological behaviors of a CNHox suspension of dispersed in ethylene glycol and reported non-Newtonian behavior. However, they examined only dilute suspensions and did not discuss the particle structure and/or dispersion−aggregation behavior. Therefore, the rheological behavior of CNHox suspensions has been poorly understood. In addition, the dependence of interaction between rough particles on salt concentration [34,35] has been reported. Nevertheless, no relationship between the suspension rheology of rough particles and rough-particle interactions affected by changes in salt concentration has been reported.

So far, systematic experiments on the rheology of suspensions of CNHox and rough particles are scarce in terms of examinations of roughness, porosity, and DLVO-like interactions. To deepen our understanding of the rheology of suspensions of CNHox, as well as our understanding of suspensions of rough and porous particles, we aimed at measuring the rheological behavior of an aqueous suspension of CNHox and discussing the results from the perspective of the relationship between suspension rheology and particle structure or aggregation−dispersion. The rheological behavior was studied via examination of shear viscosity and dynamic viscoelasticity, with different EDL interactions controlled by different salt concentrations. 

The results show that low-concentration CNHox suspensions have high viscosity. Dor example, we found a viscosity of 200 Pa∙s under low-shear conditions with a 5.0 wt.% CNHox suspension; this is almost the same viscosity found for a 60 wt.% colloidal silica suspension. These results could provide useful information to support using an CNHox suspension in industrial applications. Also, the CNHox suspensions with various salt concentrations exhibited approximately the same viscosities, even though a previous study has shown that particles are supposed to aggregate under conditions of increased salt concentration. We attribute this result to the fact that the effect of particle surface roughness on suspension rheology is greater than that of the aggregation−dispersion behaviors associated with changes in salt concentration. Such results and the discussion thereof could provide novel insights that contribute to our understanding of the effects of particle structure and dispersion−aggregation behaviors on rheology. 

## 2. Materials and Methods

### 2.1. Materials

The CNHox used was obtained from NEC corporation (Tokyo, Japan). According to the manufacturer, CNHox was prepared by oxidizing CNH in 30% H_2_O_2_ at 100 degrees Celsius for 3 h [12]. Oxidation opens the closed tips of the nanohorns and introduces carboxyl groups at the hole edges [12]. The maximum specific surface area of CNHox was reported to be 1720 m^2^/g [9]. TEM and the determination of hydrodynamic diameter by dynamic light scattering by Tian et al. [36] and Omija et al. [32] demonstrated that the CNHox particles exist as irreversible aggregates.

KCl was used to control the aggregation−dispersion behavior without changing the pH or the viscosity of the suspending medium and without charge neutralization. KCl (Wako Pure Chemical Industry, Osaka, Japan, JIS special grade) solution was prepared by dissolving KCl in deionized water (Elix 5, Millipore, Tokyo, Japan) and was used to adjust the electrolyte concentration of the suspensions. The KCl solutions were filtered using a syringe filter with a pore size of 0.20 μm (DISMIC-25HP, Advantec, Tokyo, Japan). 

CNHox suspensions were prepared by dispersing CNHox powder into water or KCl solution and shaking well. The pH of the CNHox suspensions was 5.78 (±0.53). The stability ratio of the CNHox aqueous suspension was studied by time-resolved dynamic light scattering by Omija et al. [32]. Also, the time from sample preparation to measurement, 0–48 h, did not show any significant difference. 

For comparison, silica suspensions were also prepared. Colloidal silica particles (KE-P10, Nippon Shokubai, Osaka, Japan) with diameters of 100 nm, which is almost equal to the hydrodynamic diameter of CNHox, were used. The hydrodynamic diameter of the silica particles was about 145 nm. To reduce the moisture and lower alcohol content, the silica powder was heated at 120 °C for 20 h before the suspensions were prepared. The suspensions were prepared by dispersing silica powder into water or KCl solution. The mixture was stirred by a planetary mixer (AR-100, THINKY Corporation, Tokyo, Japan) for 15 min. The pH of the silica suspensions was 6.45 (±0.45).

Before measurement, around 5 mL of each of the prepared suspensions was dispersed by ultrasonic homogenizer (CGoldenWall JP, Amazon, Tokyo, Japan) at 120 W for 10 s.

### 2.2. Rheological Measurements

**Figure 2:**
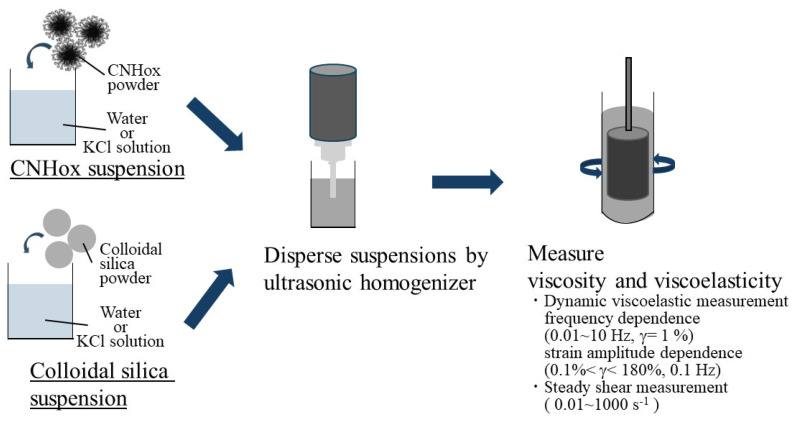
Schematic image of experimental flow.

The rheological behavior was examined using a rheometer with two co-axial cylinders (ONRH-1, Ohna Tech, Tsukuba, Japan) with a rotating outer cylinder and a stationary inner bob. The prepared suspensions were poured into the outer cylinder, which was then set to the rheometer. Once the cylinder was set and immersed in a temperature-controlled bath in the rheometer, the rheological measurements were started. The frequency dependence (frequency: 0.01~10 Hz, strain: 1%) and strain dependence (frequency: 0.01 Hz, strain 0.1~180%) of storage modulus *G′* and loss modulus *G″* and shear-rate dependence of steady-shear viscosity (shear rate: 0.01–1000 s^−1^) were measured continuously. The measurements of frequency dependency and viscosity were performed in duplicate in forward frequency sweeps or shear-rate sweeps. These measurements were carried out for the suspensions of CNHox and silica under different solid and KCl concentrations. The temperature of the temperature-controlled bath was set to 20 °C. A schematic image of the experimental flow is shown in Figure 2.

## 3. Results & Discussion

### 3.1. Rheological Behavior of CNHox Suspensions

**Figure 3:**
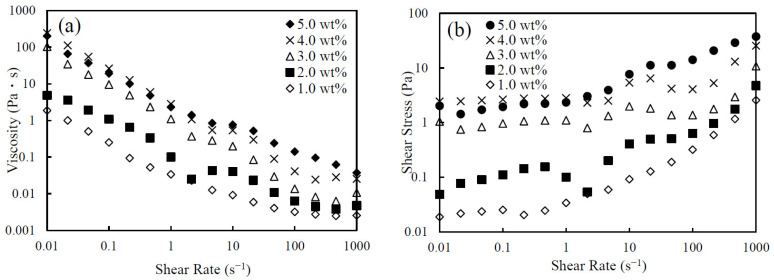
The (**a**) viscosity and (**b**) shear stress vs. shear rate of a CNHox suspensions with various concentrations of CNHox at 0 mM KCl.

Figure 3a shows the relationship between the viscosity of the CNHox suspensions and the shear rate. As shown in this figure, the viscosity increases in accordance with the particle concentration. In all cases, the suspensions exhibit shear-thinning behavior. That is, the viscosity decreases as shear rate increases. Figure 3b shows the relationship between the shear stress and the shear rate of the CNHox suspensions. The figure demonstrates that the shear stress reaches an almost constant value at lower shear rates and starts increasing above a shear rate of 1 s^−1^ for the suspensions with solid concentrations greater than 3.0 wt.%. These constant values are considered to represent yield stress. The presence of yield stress indicates the formation of structures at low shear rates. Also, the decrease in viscosity with increasing shear rate is probably due to the collapse of the structures formed at low shear rates [37]. At low salt concentrations, a structure like a colloid crystal would melt as the shear rate increased. At low salt concentrations and higher shear rates, the formed aggregate would be broken apart and the effective volume fraction might decrease.

**Figure 4:**
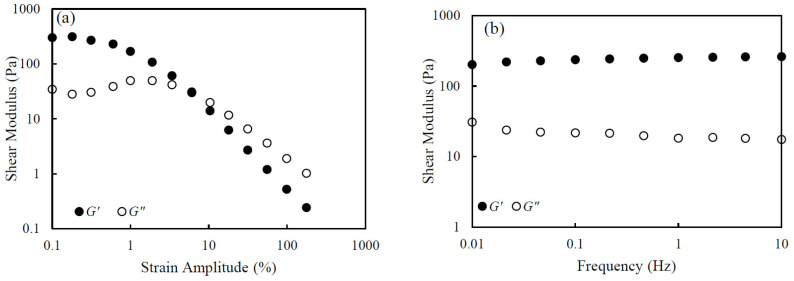
The storage modulus *G′* and loss modulus *G″* of 5.0 wt.% CNHox suspension at 0 mM KCl (**a**) vs. strain amplitude at 0.01 Hz frequency and (**b**) vs. frequency at 1% strain amplitude.

In Figure 4a, the storage modulus G′ and loss modulus G″ of the 5.0 wt.% CNHox suspension are plotted as a function of strain amplitude γ at a frequency of 0.01 Hz. The figure shows that G′ and G″ remain almost constant at values of γ below 1%. As the strain increases over 1%, G′ decreases monotonically and G″ increases slightly and then decreases. Above 10% strain, G′ becomes smaller than G″ and both G′ and G″ decrease monotonically. Generally, the reduction in G′ is considered to represent yield, with the sample starting to soften or deform under strain. Also, the cross-over point of G′ and G″ represents the strain amplitude at which the sample starts to flow. These results mean that the gel-like structure of a CNHox suspension is developed at lower strains and that the suspension then starts to deform and flow as strain amplitude increases. These results also agree with the results of steady-shear viscosity, which show both yield stress and shear-thinning.

Figure 4b shows the storage modulus G′ and loss modulus G″ plotted against frequency for the 5.0 wt.% CNHox suspension at a strain amplitude of 1%. This strain amplitude is in the range of the liner viscoelastic (LVE) region, where G′ is almost constant (Figure 4a). The results for both G′ and G″ demonstrate virtually no dependence on frequency. In addition, G′ is greater than G″ over the entire range of frequency. This behavior is typical of a gel-like elastic response in colloidal suspensions. The results agree with the results of steady shear measurement, showing yield stress.

### 3.2. Effects of Particle Structure on Suspension Viscosity

The rheological features of CNHox suspensions affected by the structure of particles such as porousness can be clarified based on the difference between the rheology of a CNHox suspension and that of a suspension of solid spherical silica particles.

**Figure 5:**
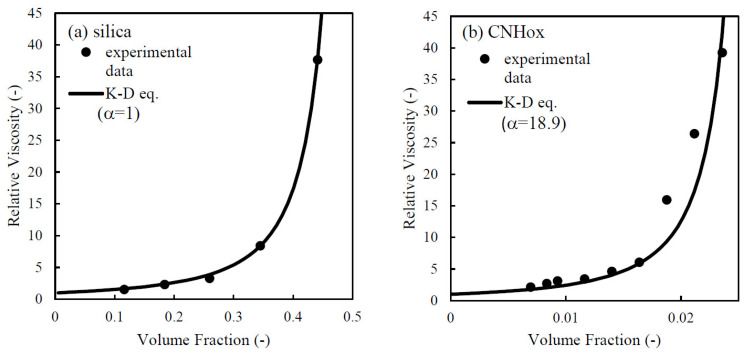
The relative viscosity $\eta_{r}\;$ plotted against the volume fraction of (**a**) silica suspensions and (**b**) CNHox suspensions with 10 mM KCl at 1000 s^−1^. The filled circles denote experimental data. The volume fractions were calculated by using the particle densities of graphite (=2.18 g/cm^3^) from ref. [38] and silica (=1.9 g/cm^3^) by the manufacture. The solid line is the plot of the K−D equation with ϕ_max_ = 0.527, α = 1 for silica and α = 18.9 for CNHox.

Figure 5 shows the relative viscosity $\eta_{r}$ and the suspension viscosity $\eta_{s}$ divided by the medium viscosity $\eta_{0}$, each plotted against the volume fraction of particles at a high shear rate of 1000 s^−1^ for Figure 5a silica suspensions and Figure 5b CNHox suspensions with 10 mM KCl. The symbols denote experimental data. The reasons for choosing this shear rate and KCl concentration are as follows. At this shear rate, the effect of Brownian motion is thought to be small. At this salt concentration, EDL interaction and aggregation are thought to be small, as discussed in Section 3.3. Therefore, at this concentration and shear rate, we could focus on the effect of particle structure because other factors such as Brownian motion, EDL interaction, and aggregation are negligible. As shown in this figure, the relative viscosity increases in a power-law manner with increasing particle volume fraction. However, the fit of the Krieger−Dougherty equation to the data in Figure 5 is better for silica than for CNHox. The equation is for a suspension of spheres. Thus, the fit is not good for CNHox, which has a rough structure. Furthermore, the CNHox suspensions show greater values of viscosity at lower volume fractions compared to the silica suspensions. This greater viscosity is probably because of the large effective volume of CNHox, which results from its porous structure.

To quantitatively evaluate the effective volume of an CNHox particle, the following calculations were done. The effective volume of CNHox here is the apparent volume, which includes the volume of solid CNHox and the volume of the pores in the CNHox aggregates. Firstly, the particle volume fraction in suspension was obtained from the mass concentration by using the graphene density of 2.18 g/cm^3^ [38] for CNHox and the silica density of 1.9 g/cm^3^. Secondly, we used the Krieger−Dougherty (K−D) equation for the relationship between the relative viscosity and the volume fraction of suspension of rigid spherical particles ϕ  with fitting parameters α and *ϕ*_max_, as follows:

(1) $$\eta_{r}=\frac{\eta_{s}}{\eta_{0}}={\left(1-\frac{\alpha \phi }{\phi_{max}}\right)}^{-2}$$

The values of α and *ϕ*_max_ were extracted from the comparison of the K−D equation with the experimental data. Here, α is an effective-volume factor. The effective volume fraction was obtained by multiplying the volume fraction by α. The K−D equation was fitted with the data from the silica suspension with α = 1 by adjusting the value of *ϕ*_max_ before fitting with the CNHox data. As a result, it was decided that *ϕ*_max_ = 0.527 for the silica suspension. Then, the experimental data for the CNHox suspension was fitted with the K−D equation with *ϕ*_max_ = 0.527 by adjusting the value of α. When α = 18.9, the K−D equation agrees well with the experimental data for CNHox. This agreement with α = 18.9 means that the effective volume fraction of CNHox is about 18.9 times larger than that of a hard spherical particle of the same volume. This large effective volume is probably the effect of the solvent being present in the horn or between horns in aggregates, where it behaves as a part of the particle. Consequently, the effective volume fraction of the CNHox particle becomes large in suspension and the viscosity also becomes high. Through this calculation, the high viscosity, which is one of the major rheologic features of a CNHox suspension, could be quantitatively expressed in terms of the effective volume for the first time.

However, there is a possibility of overestimating the effective volume. The finding that the effective volume is 18.9 times larger than that of hard spherical particles was calculated under the assumption that the high viscosity is entirely caused by the large effective volume of CNHox particles. However, suspensions of particles with rough surfaces also show high viscosity [20,21], and this analysis can not separate the contributions to viscosity from the particle surface roughness and the large effective volume fraction. Thus, the actual effective volume of CNHox could be smaller than the value of 18.9 times that of a solid spherical particle.

### 3.3. Effect of Salt Concentration on Rheological Behavior

**Figure 6:**
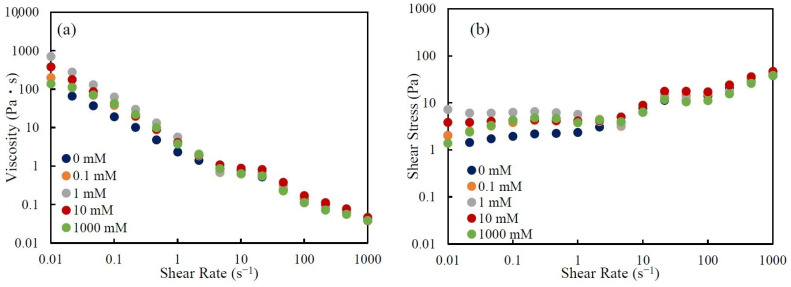
The (**a**) viscosity and (**b**) shear stress vs. shear rate of 5.0 wt.% CNHox suspensions at various KCl concentrations. Different colors denote different KCl concentrations.

Figure 6 shows the (a) viscosity and (b) shear stress vs. shear rate for CNHox suspensions of 5.0 wt.% with various KCl concentrations. The viscosity and shear stress of a CNHox suspension show almost no dependence on KCl concentration. The values of the viscosity and shear stress are similar irrespective of KCl concentration, especially under high shear rate. Also, all the suspensions show shear-thinning and yield stress.

**Figure 7:**
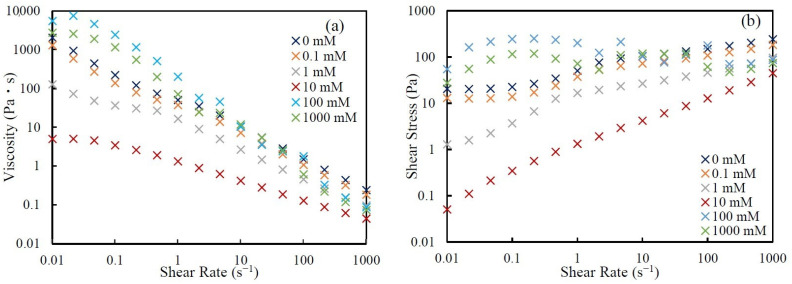
The (**a**) viscosity and (**b**) shear stress vs. shear rate of 60 wt.% silica suspension with various KCl concentrations. The different colors denote different KCl concentrations.

Figure 7 shows the (a) viscosity and (b) shear stress vs. shear rate of 60 wt.% silica suspensions with various KCl concentrations. The viscosity and the degree of shear-thinning behavior were strongly affected by the change in KCl concentration. For example, the values of viscosity of the silica suspensions at low shear rates with 0 mM and 0.1 mM were about 1000 Pa∙s. The viscosity of silica suspension with 1 mM KCl decreased slightly, to 100 Pa∙s, and the addition of 10 mM KCl decreased the viscosity to 5 Pa∙s. The silica suspension with 10 mM KCl showed the lowest viscosity among all six suspensions with different salt concentrations. For the suspensions with 100 mM and 1000 mM KCl, the viscosity became larger than those of suspensions with 0 mM and 0.1 mM KCl, especially at low shear rates. As a result of the change in viscosity at such low shear rates, the degree of shear-thinning behaviors also became different. This is because the effect of change in KCl concentration is smaller at high shear rates. Additionally, the shear stress also depends on the salt concentration. For example, the suspensions with 0 mM and 0.1 mM KCl showed clear yield stress, but the suspensions with 1 mM and 10 mM KCl did not show yield stress. The suspensions with 100 mM and 1000 mM KCl showed almost constant shear stress over the whole range of shear rates.

Nakamura et al. [28] also showed the salt-concentration dependence of the viscosity of the silica suspension. They concluded that the dependence of viscosity on salt concentration is induced by the configuration of silica particles in suspension at low shear rates. The particle configuration might be changed by the addition of salt because the balance of the EDL repulsive force and the van der Waals attractive force changes with salt concentration. At low salt concentrations, the particles form an ordered structure because of the strong and long-range EDL repulsion; thus, the viscosity at low shear rates is high. The addition of KCl weakens the EDL repulsion; as a result, the ordered structure becomes imcomplete and the viscosity at low shear rates decreases. Further increases in KCl concentration eliminate the EDL repulsion, and the remaining attractive force becomes dominant. As a result, aggregation occurs and the viscosity at low shear rates increases again. Such structures formed at low shear rates are broken by shear; therefore, the dependence of viscosity on KCl concentration becomes small at high shear rates. Our results for the silica suspensions also suggest that such changes in the configuration of silica particles in suspension might be caused by changing salt concentrations and might affect suspension rheology.

**Figure 8:**
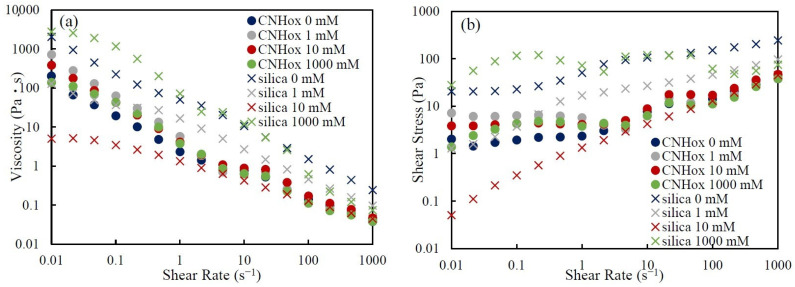
The (**a**) viscosity and (**b**) shear stress vs. shear rate of 5.0 wt.% CNHox suspensions and 60 wt.% silica suspensions with various KCl concentrations. The compared particle-mass concentrations are thought to be almost the same effective volume concentration discussed in Section 3.2. The circles denote CNHox suspensions, and the crosses denote silica suspensions. The different colors denote different KCl concentrations.

In Figure 8, the (a) viscosity and (b) shear stress vs. shear rate of the CNHox suspensions and the silica suspensions with various KCl concentrations are plotted together. Compared to that of silica-suspension rheology, the salt-concentration dependence of CNHox-suspension rheology is extremally weak.

According to Omija et al. [32], the aggregation−dispersion behavior of CNHox in dilute aqueous salt solutions follows the DLVO theory. Thus, the rheology of a CNHox suspension is also affected by the change in salt concentration because of the change in particle configuration in suspension, as observed for the silica suspension. However, the viscosity of CNHox suspensions shows weak dependence on salt concentration. We consider that this contradiction is the result of the presence of irreversible dahlia-like aggregates of CNHox and especially the result of their surface roughness.

**Figure 9:**
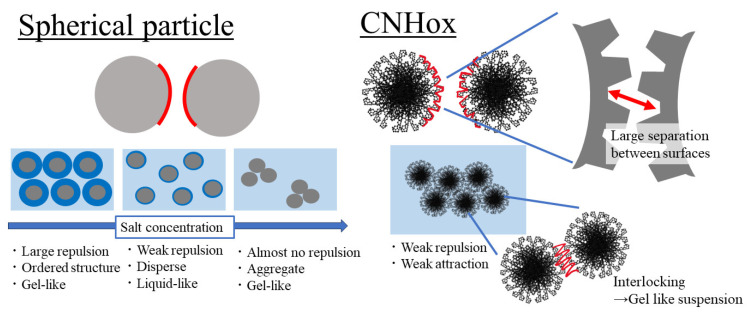
Schematic image showing reasons for the weak dependence of the rheology of a CNHox suspension on salt concentration.

We show a schematic image explaining the weak dependency of the viscosity of a CNHox suspension on salt concentration in Figure 9. The effective distance between particles with surface roughness, like CNHox, is thought to be greater. Since the surface has asperities, the distance between hollow areas is greater. Therefore, interactions such as the EDL force or vdW force between particles with surface roughness are weak compared to those between spherical particles. Indeed, Bhattacharjee et al. [34] reported that the repulsion between rough surfaces is attenuated. Also, Considine et al. [35] reported that vdW force between rough surfaces becomes weak due to the particles’ small radius of curvature. The surface of a CNHox aggregate can be regarded to be rough. Therefore, the interaction between CNHox particles would be weakened due to the particle structure. In a concentrated suspension such as a CNHox suspension of 5.0 wt.%, the particles can approach each other under a weak repulsive force. The horns sticking out from the particle surface cause interlocking, and the suspensions show gel-like behavior regardless of changes in salt concentration. These results and discussion above systematically elucidate the rheological features of suspensions of rough particles and the interaction between rough particles under varied salt concentrations.

Also in Figure 8, for the CNHox suspensions, the inflection points can be seen at shear rates of around 3–5 s^−1^. Such inflections are not obvious for silica suspensions. Therefore, the appearance of inflection may be inherent to the CNHox suspensions. At this moment, the distinct mechanisms underlying this inflection are not clear. Probably, a reconfiguration of CNHox aggregates induces this inflection.

**Figure 10:**
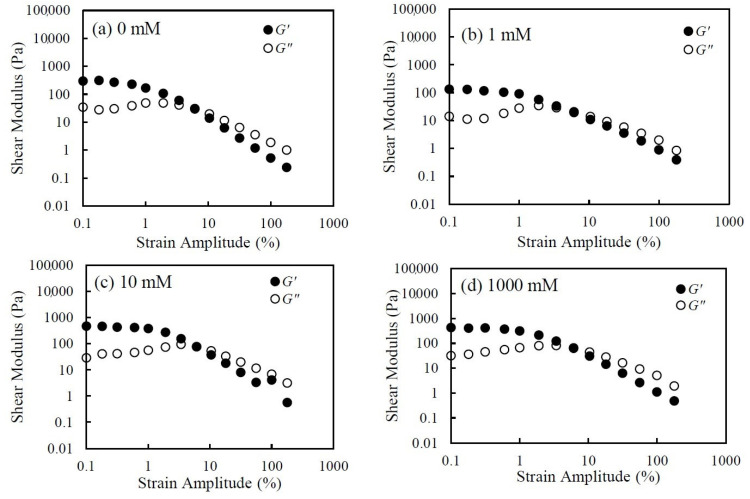
The storage modulus *G′* and loss modulus *G″* vs. strain amplitude at 0.01 Hz of 5.0 wt.% CNHox suspensions with various KCl concentrations of (**a**) 0 mM, (**b**) 1 mM, (**c**) 10 mM, and (**d**) 1000 mM.

Figure 10 shows the measured viscoelasticity data for CNHox suspensions at various salt concentrations under the strain amplitude sweep. The viscoelasticity has weak dependence on salt concentration. For example, the G′ and G″ values are almost the same, even when the salt concentration is different. Additionally, the strain amplitudes where the G′ and G″ cross over are also almost the same regardless of salt concentration. Again, this result shows weak dependence on salt concentration, consistent with the viscosity and shear-stress results.

**Figure 11:**
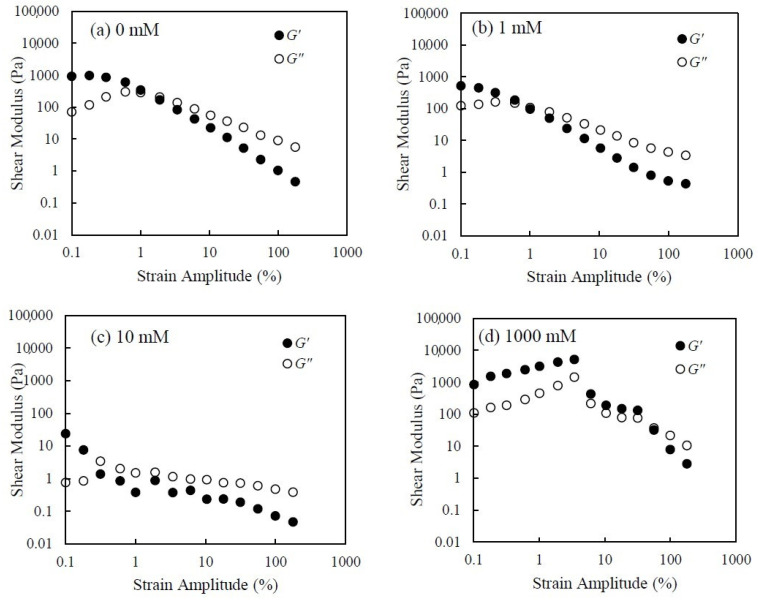
The storage modulus *G′* and loss modulus *G″* vs. strain amplitude at 0.01 Hz of 60 wt.% silica suspensions with various KCl concentrations of (**a**) 0 mM, (**b**) 1 mM, (**c**) 10 mM, and (**d**) 1000 mM.

Figure 11 shows the viscoelastic data for silica suspensions under strain amplitude sweep tests obtained at different salt concentrations. The viscoelasticity of silica suspensions depends strongly on salt concentration. For the suspension with 0 mM KCl, the G′ shows a liner viscoelastic region (LVE) at a strain amplitude lower than 1%. Then, the G′ and G″ cross over around the strain amplitude of 1%. As for the suspension with 1 mM KCl, both the G′ and G″ are a little bit lower than those for 0 mM sample. Also, the decrease in G′ became faster as the strain amplitude increased, and the strain amplitude where G′ and G″ cross over is lower compared to that of the suspension with a lower salt concentration. As for the suspension with 10 mM KCl, the values of G′ and G″ are much lower than those of other samples, and the changes in G′ and G″ with increasing strain amplitude shows different patterns. These results indicate that the suspension with 10 mM KCl is more liquid-like and flows more easily. As for the suspension with 1000 mM KCl, G′ increases with increasing strain amplitude to around 5% and then starts to decrease. The value of G′ and G″ cross over around 50% of strain amplitude. This result means that the silica suspension with 1000 mM KCl is gel-like and that a large strain amplitude is needed to break down the network and allow flow. These results mean that the silica suspension becomes gel-like with increasing salt concentration, and this result agrees with the viscosity and shear-stress results.

## 4. Conclusions

This research experimentally examined the rheological behavior of aqueous suspension of CNHox and discussed the rheological behavior from the perspective of the structure and aggregation−dispersion behavior of CNHox. The suspensions of CNHox with particle concentrations over 3.0 wt.% showed gel-like behavior under low shear rates or low strain amplitudes. With increasing shear or strain amplitude, the suspensions became liquid-like. Also, the CNHox suspension showed higher viscosity with smaller particle mass concentration compared to suspensions of solid and spherical silica particles because of the porous structure of CNHox. The estimated effective volume of CNHox calculated by using the K−D equation was 18.9 times larger than that calculated with the mass concentration and density. Moreover, the rheological behavior of a CNHox suspension showed weak dependence on salt concentration, while the colloidal stability of dilute suspension of CNHox follows the DLVO theory. The difference seen in the rheology and colloidal stability of CNHox may be due to the surface roughness of CNHox particles.

The CNHox suspension has the potential to be used as an electrochemical capacitor, thermal fluid, and so on due to its unique structure, high conductivity, high thermal conductivity, and high specific surface area. The rheological properties of a CNHox suspension shown in this research can be used to improve the efficiency of its utilization for the various applications listed previously.

## Data Availability

The data are available from the authors upon reasonable request.
